# Imaging Diagnosis of Desmoplastic Small Round Cell Tumor: A Report of Two Cases

**DOI:** 10.7759/cureus.58037

**Published:** 2024-04-11

**Authors:** Jonathan Pimiento Figueroa, Mónica Royero-Arias, Marcia Mejia, Elkin E Garcia

**Affiliations:** 1 Radiology, Servicios de Salud San Vicente Fundación, Medellín, COL; 2 Pediatric Radiology, Servicios de Salud San Vicente Fundación, Medellín, COL; 3 Radiology, Universidad de Antioquia, Medellín, COL

**Keywords:** imaging diagnosis, soft-tissue sarcoma, peritoneal malignancy, cross-sectional imaging, intra-abdominal neoplasms, desmoplastic small round blue cell tumours

## Abstract

Desmoplastic small round cell tumor (DSRCT) is a rare multifocal peritoneal sarcoma, typically found in adolescent and young adult males. Symptoms are nonspecific and vary depending on tumor involvement. Diagnosis is primarily histopathological, although imaging results can assist in the diagnostic process. Although not pathognomonic, certain radiologic findings can help narrow down potential diagnoses and sometimes suggest the condition, as seen in our cases. Treatment options are not well-established or effective, and despite employing various therapeutic approaches, the prognosis remains poor. We present two cases of boys aged 11 and 10 with a final diagnosis of DSRCT, emphasizing the imaging findings.

## Introduction

Desmoplastic small round cell tumor (DSRCT) is an uncommon peritoneal sarcoma with aggressive behavior and a bleak prognosis; its name reflects some of the main histological features such as nests of small round cells with desmoplastic stroma. It predominantly impacts White/Hispanic male adolescents and young adults [1,2]. Initially documented in 1989 by Gerald et al. [3], medical records have documented around 450 cases of DSRCT up to the year 2019 [2].

Symptoms related to DSRCT are often nonspecific; this is the primary reason why early diagnosis is frequently challenging with diagnosis algorithms focusing on imaging findings and ultimately on pathological results [1,2,4]. Common imaging findings include one or multiple peritoneal masses without a clear organ of origin, ascites, intraabdominal lymphadenopathy, and sometimes distant metastases [2,4,5]. The prognosis is generally unfavorable despite various available treatment options such as surgery, radiotherapy, and chemotherapy [2,4].

## Case presentation

Case 1

**Figure 1:**
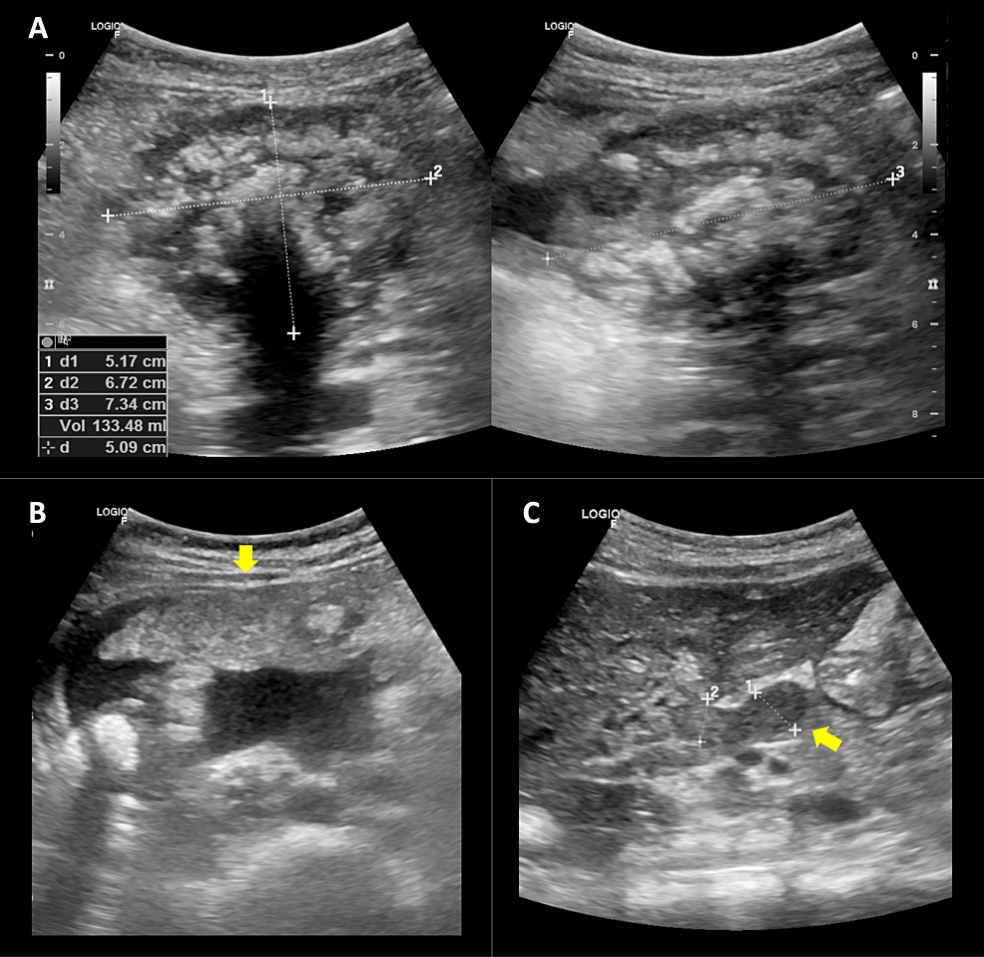
Ultrasound A, located in the mesogastrium, the dominant mass appears heterogeneous with poorly defined edges and some hyperechoic areas causing a posterior acoustic shadow, measuring up to 7.3 cm; B, thickening and increased echogenicity of the omentum (arrow) associated with moderate ascites; C, periportal lymphadenopathy of up to 14 mm (arrow)

An 11-year-old male patient, with an unremarkable medical background, presented with a one-month duration of self-resolving diffuse abdominal discomfort exacerbated postprandially and associated with hyporexia, constipation, and bladder tenesmus. Following a thorough assessment, the emergency department conducted an abdominal ultrasound examination, which disclosed an abdominal mass located in the mesogastrium of uncertain origin, in addition to peritoneal thickening, ascites, and lymphadenopathy in the hepatic hilum (Figure 1).

**Figure 2:**
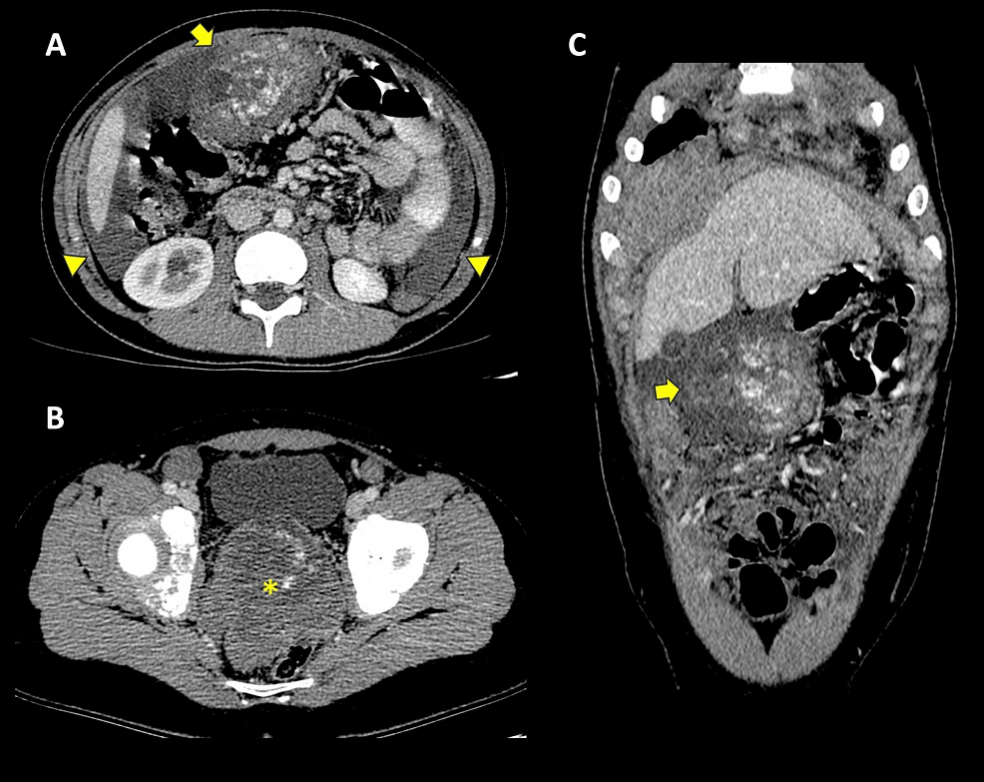
Contrasted tomography A-C, axial and coronal, shows a diverse dominant mass in the greater omentum/epigastrium with dense calcifications of uncertain solid organ origin (arrows), another mass with similar characteristics in the rectovesical space (asterisk) along with ascites (arrowheads).

**Figure 3:**
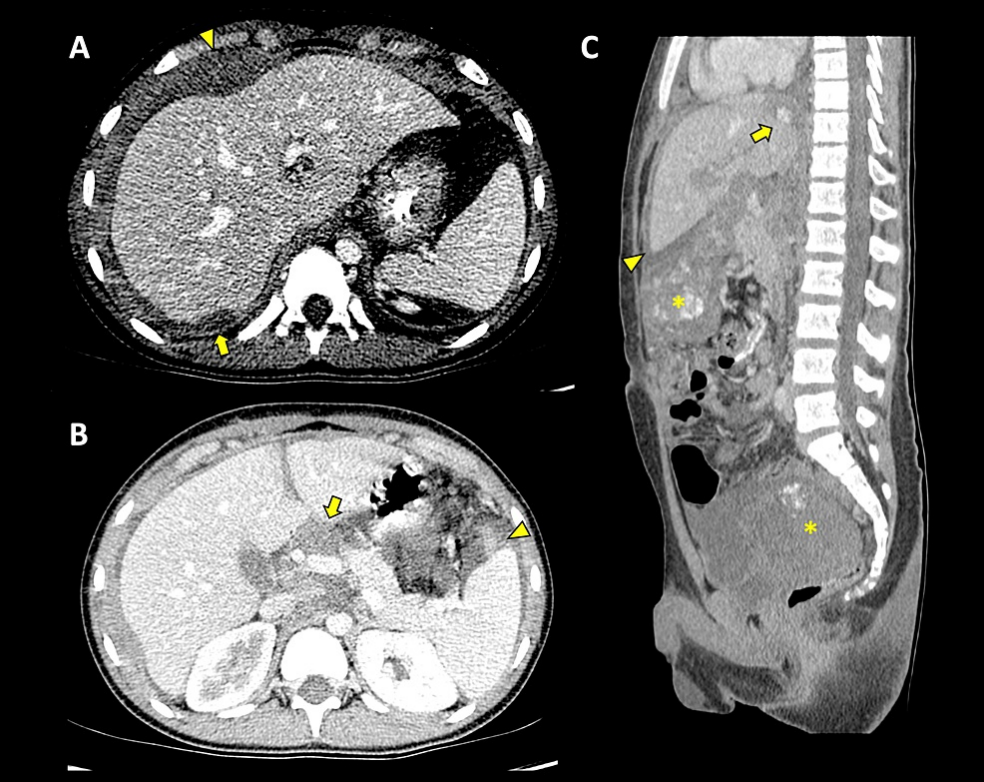
Contrasted tomography A, axial, nodular thickening of the right hemidiaphragm compromises the diaphragmatic pillar (arrow) and the anterior aspect (arrowhead) due to metastatic involvement; B, axial, periportal lymphadenopathy (arrow), and ascites (arrowhead); C, sagittal, nodular involvement of the right diaphragmatic crus (arrow), also showing dominant masses in the greater omentum/epigastrium (asterisk) and rectovesical space (asterisk) and ascites (arrowhead)

Subsequently, a contrast-enhanced tomographic study of the thorax and abdomen revealed multiple heterogeneous peritoneal masses with calcifications and cystic/necrotic areas. The largest masses were found in the greater omentum (epigastrium) and the rectovesical space, measuring up to 7.3 cm (Figure 2). Lymphadenopathy was observed in the mesentery, retroperitoneum, and hepatic hilum. Nodular thickening of the right hemidiaphragm due to metastatic involvement was also noted (Figure 3). Additionally, there was secondary dilation of the right urinary tract due to extrinsic compression of the distal ureter.

**Figure 4:**
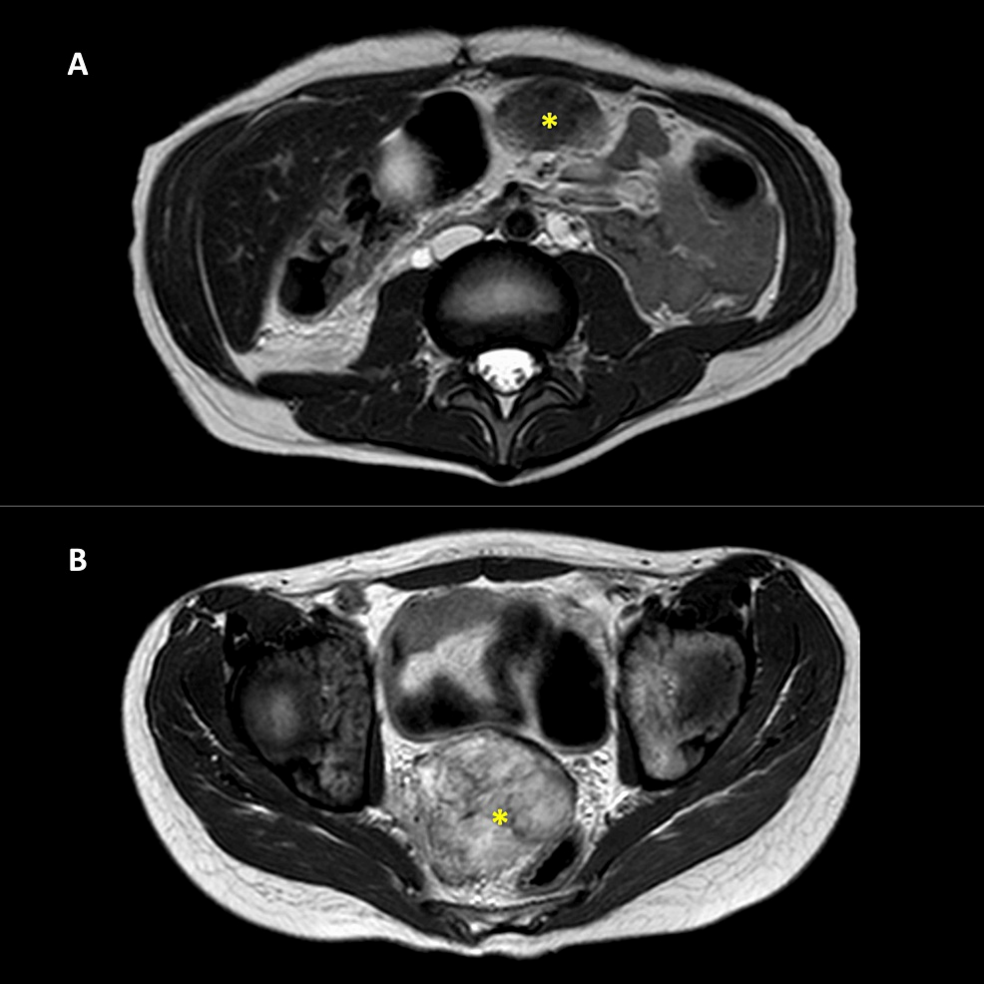
Contrasted magnetic resonance (follow-up) Axial T2 sequences. Larger masses (asterisks) in the greater omentum/epigastrium (A) and rectovesical space (B), with a decrease in size compared to the initial tomographic study.

Based on imaging findings, a diagnosis of peritoneal sarcoma vs desmoplastic small round cell tumor was made. An open biopsy confirmed the diagnosis of DSRCT. Chemotherapy was started, resulting in a partial response (Figure 4). Unfortunately, the patient died nine months after diagnosis during follow-up.

Case 2

**Figure 5:**
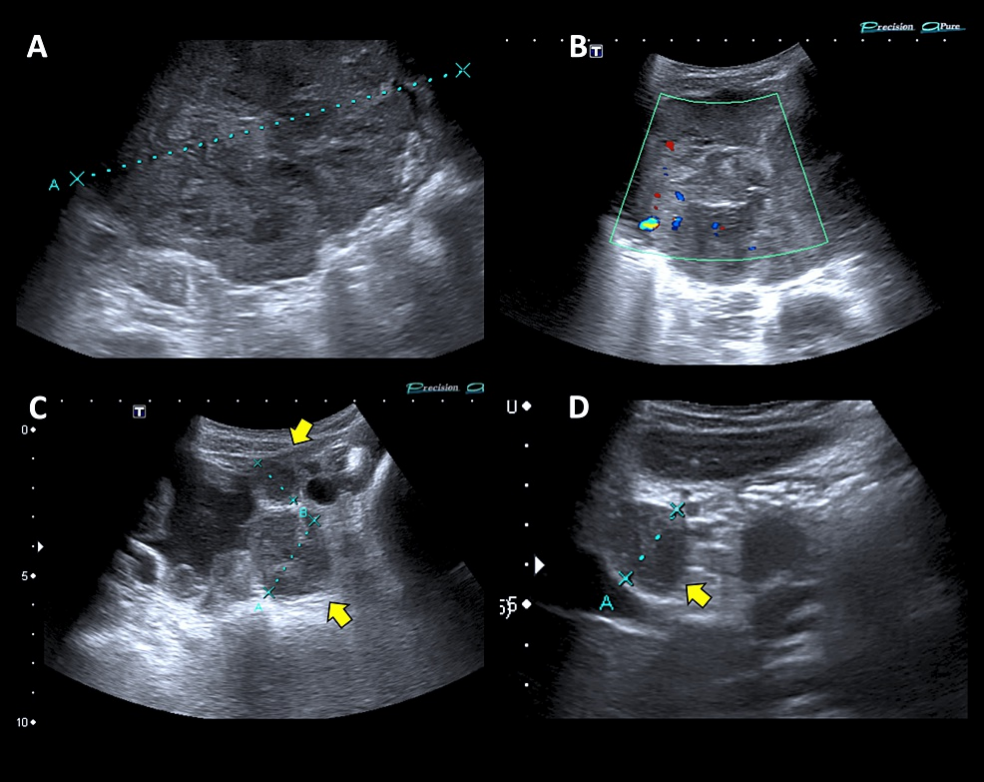
Ultrasound A, dominant mass is present on the right side of the abdomen with central vascularization and a diameter of up to 13 cm; C-D, multiple smaller peritoneal implants are found in the lower quadrants (arrows)

A 10-year-old boy with a background of type 1 diabetes mellitus presented with right-sided chest pain that was pleuritic and self-limited. During the exam, an unexpected abdominal mass was discovered, with no other accompanying symptoms. An abdominal ultrasound was requested in the emergency department, revealing several intra-abdominal masses; the biggest one being in the right iliac fossa and measuring up to 13 cm, along with ascites (Figure 5).

**Figure 6:**
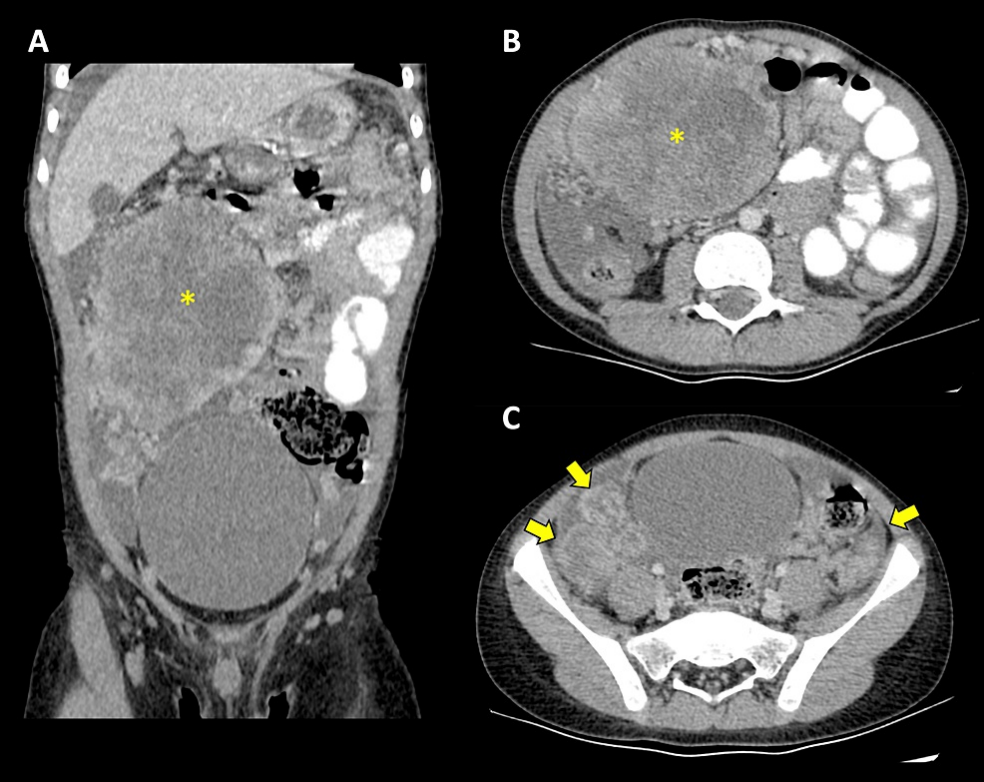
Contrasted tomography A, coronal; B-C, axial. Dominant mass in the right quadrants of the abdomen with necrotic/cystic changes (asterisk) and smaller peritoneal implants (arrows) affecting the lower quadrants.

Following this, a contrasted tomographic examination of the thorax and abdomen revealed numerous masses and peritoneal implants affecting the omentum, the root of the mesentery, the parieto-colic leaks, and the lower quadrants (Figure 6). The main mass is situated at the root of the mesentery on the right side, measuring up to 12 cm in diameter, displaying central necrotic alterations but no calcifications (Figure 6). There is no extraperitoneal extension.

Based on the tomographic findings, a peritoneal tumor diagnosis was established, suggesting the following potential diagnoses: desmoplastic small round cell tumor, rhabdomyosarcoma, or lymphoma. An ultrasound-guided biopsy of the main lesion was conducted, revealing pathological results confirming DSRCT. The patient died about three months post-diagnosis.

## Discussion

Desmoplastic small round cell tumor (DSRCT), initially outlined in 1989 by Gerald et al., is an exceptionally uncommon form of peritoneal sarcoma [3]. As of 2019, there have been reports of up to 450 cases in the literature [2]. DSRCT is classified within the group of small round cell malignancies, such as neuroblastoma, Wilms tumor, Ewing sarcoma, malignant lymphoma, rhabdomyosarcoma, anaplastic synovial sarcoma, and primitive neuroectodermal tumor [6].

DSRCT primarily affects adolescents and young adults, with a mean age of about 20 years, mostly in males, with a ratio of up to 10.75:1 [2]. Symptoms at diagnosis are typically nonspecific and linked to the affected anatomical site. These symptoms may include abdominal pain, constipation, a feeling of an abdominal mass, weight loss, urinary symptoms, and lower back pain [7].

Imaging findings in the literature are primarily based on tomographic results [2]. The key intra-abdominal discovery is the existence of numerous soft tissue masses or nodules with no clear origin from a solid organ [2,4,8]. Among multiple abdominal masses, the largest or most prominent ones are typically located retrovesically or rectouterinely and in the peritoneal or omental region [4]; masses larger than 10 cm in diameter usually exhibit central necrotic changes [4]. Calcifications have been observed in 13-29% of cases [2,4].

The spread patterns of DSRCT involve contiguity, as well as hematogenous and lymphatic pathways [4]. Extraperitoneal areas affected may include the retroperitoneum, liver, diaphragm, lungs, bones, pleura, pancreas, kidney, and spleen [2,4,9]; among these, retroperitoneal, extra-abdominal nodal, diaphragmatic, and hepatic involvement are more common [2]. Other findings reported may include secondary hydronephrosis, ascites, and pleural effusion, depending on the extent of local and metastatic involvement [2-4,10].

The diagnosis of DSRCT is typically made based on histological findings showing well-defined nests of small, round cells with abundant desmoplastic stroma [11]. Immunohistochemistry helps distinguish it from other small and round cell tumors, with the most specific diagnosis being the chromosomal translocation (t11;22) (p13;q12); this mutation leads to a formation of the EWSR1-WT1 fusion oncogene [11].

To date, there is still no effective therapeutic strategy, with reported five-year survival rates between 4% and 18% despite aggressive therapeutic management [12]. Among the various therapeutic approaches, such as surgery, radiotherapy, chemotherapy, and intraperitoneal hyperthermic perfusion (HIPEC), the latter shows promise in treating DSRCT, achieving a mean survival rate of up to 71% at three years. In DSRCT, HIPEC has been described as heated cisplatin at a dose of 100- 150 mg/m^2^ after surgical cytoreduction [13]. Nevertheless, it is crucial to emphasize that despite multimodal treatment, further studies and research are needed to formulate an appropriate therapeutic approach [12].

## Conclusions

DSRCT is a rare neoplasm that mostly affects young White/Hispanic males. Imaging shows multiple intraperitoneal soft tissue masses/nodules with uncertain organs of origin, with the largest lesions in the retrovesical/rectouterine or peritoneal spaces. Diagnosis is typically confirmed by histopathological findings but can also be suggested by imaging results, as shown in our cases. Recognizing these findings and including this condition in the list of potential diagnoses for peritoneal masses in adolescents and young adults is crucial. The prognosis remains poor despite varied treatments, highlighting the need for further research to improve outcomes.
